# Scanner‐agnostic dynamic jaw motion generation from virtual static excursive records using open‐source Python‐based artificial intelligence (AI) interpolation

**DOI:** 10.1111/jopr.70051

**Published:** 2025-11-14

**Authors:** Mohamed Sherif Omar, Chao‐Chieh Yang, Dean Morton, Wei‐Shao Lin

**Affiliations:** ^1^ Department of Prosthodontics Indiana University School of Dentistry Indianapolis Indiana USA; ^2^ Department of Prosthodontics, Advanced Education Program in Prosthodontics Indiana University School of Dentistry Indianapolis Indiana USA; ^3^ ITI Scholarship Center, Center for Implant, Esthetic and Innovative Dentistry, Department of Prosthodontics Indiana University School of Dentistry Indianapolis Indiana USA

## Abstract

This technique describes a scanner‐agnostic digital workflow for generating dynamic mandibular motion from static virtual interocclusal records using a custom artificial intelligence (AI) algorithm and user interface. Virtual records of mandibular positions, including maximum intercuspation, protrusion, and lateral excursions, were captured with an intraoral scanner and processed through a custom interface developed using Python, an open‐source, script‐based programming language. The program interpolates intermediate positions using quantified point tracking and exports a motion path file compatible with dental computer‐aided design software. By leveraging AI and open‐source tools, this method offers a cost‐effective, non‐vendor‐specific solution for integrating individualized jaw motion into digital prosthodontic workflows.

Intraoral scanners (IOS) can generate accurate digital scans for prosthetic computer‐aided design (CAD) workflows.^1^, ^2^, ^3^ IOS can also reliably capture static virtual occlusal records representing maxillomandibular relationships.^4^, ^5^, ^6^, ^7^ Despite advancements in digital prosthodontics, most current workflows emphasize static occlusal relationships. While many CAD software platforms offer virtual articulator functionalities, these simulations rely on arbitrary model mounting and average anatomical parameters rather than patient‐specific mandibular motion data.^8^, ^9^, ^10^ As a result, prosthetic restorations frequently require clinical occlusal adjustments to accommodate functional mandibular excursions.

Jaw motion tracking systems have been developed to record and analyze mandibular dynamics.^11^ Diagnostic systems, such as electronic axiography devices (Cadiax; GAMMA Medizinisch‐Wissenschaftliche Fortbildungs‐GmbH), 3D jaw tracking systems (JT‐3D; BioResearch Associates), and jaw motion analysis platforms (K7x; Myotronics), record mandibular movement for condylar inclination assessment and axio‐pantographic tracing.^12^ However, these platforms typically do not support exporting motion data in file formats compatible with widely used CAD software.^13^ Other systems, such as digital jaw tracking devices with proprietary CAD integration (Arcus Digma II; KaVo Dental GmbH), permit data export but limit integration to proprietary CAD platforms. More recent open systems, including photometric jaw tracking devices (such as Cyclops; Zebris Medical GmbH or ModJaw; ModJaw SAS), ultrasound, and infrared optical‐based devices (JMA; Zebris Medical GmbH), allow for the capture, analysis, and export of individualized mandibular movement data for use in external CAD software.^13^ Nevertheless, the clinical application of these systems remains limited due to high equipment costs and workflow complexity.

When vendor‐specific or device‐specific solutions are needed, clinical applications are more challenging. A scanner‐agnostic technique to generate or record dynamic mandibular motion would allow clinicians to benefit from the advantages of using dynamic motion information, independent of the intraoral scanner's manufacturer. With the development of computer programming and artificial intelligence (AI) algorithms, researchers have started to develop device‐agnostic clinical applications.^14^, ^15^ This technique aimed to create a scanner‐agnostic digital workflow for generating dynamic mandibular motion from static virtual interocclusal records, using an open‐source, Python‐based AI algorithm and user interface. It enables the export of motion paths compatible with dental CAD software. The exported motion file can be freely used to design prostheses, demonstrating the practicality of the technique.

## TECHNIQUE

**FIGURE 1:**
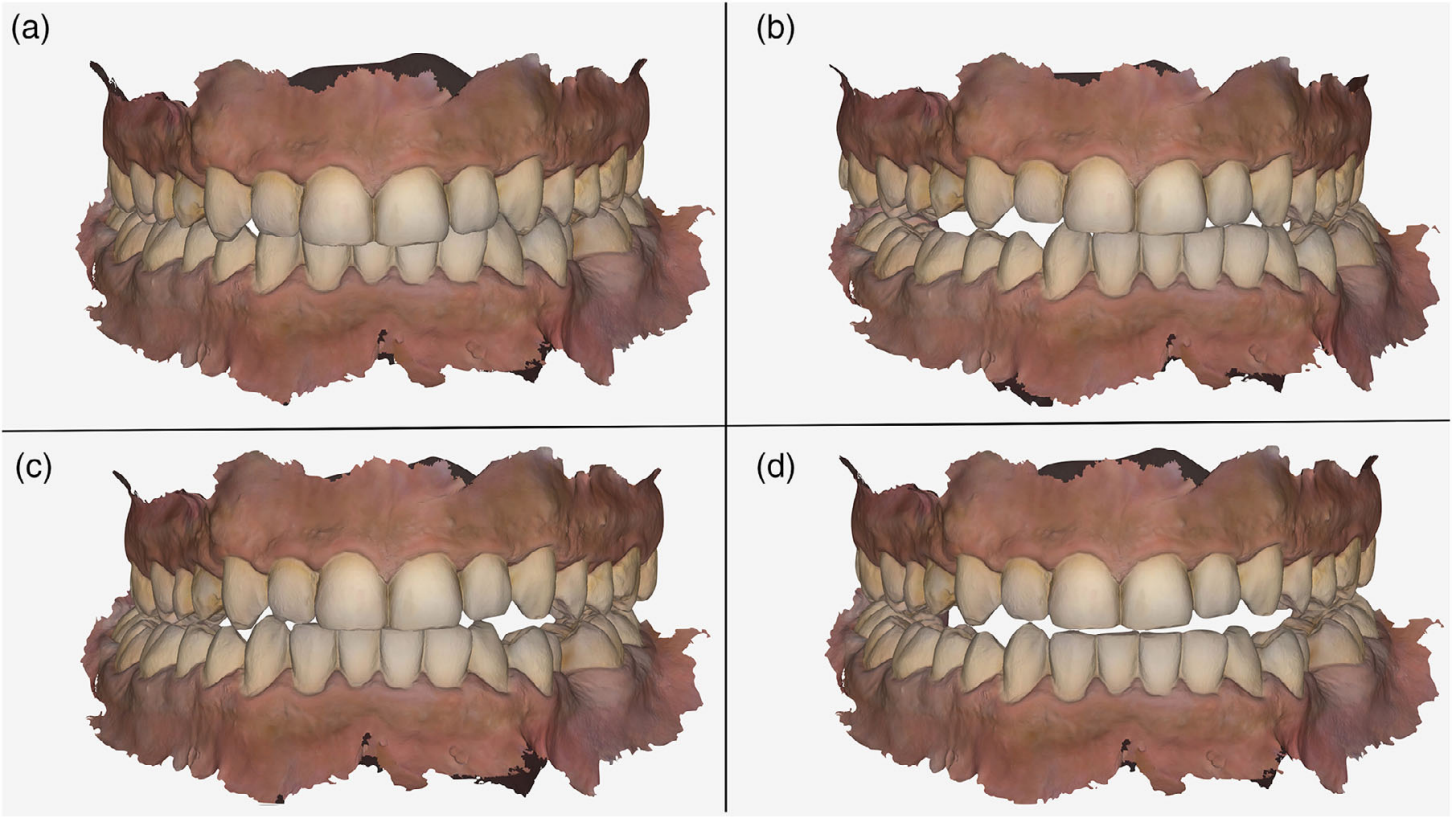
Intraoral scans of the maxillary and mandibular arches were duplicated three times and registered at different interocclusal positions: (a) maximum intercuspation position; (b) left excursion; (c) right excursion; (d) protrusion.

**FIGURE 2:**
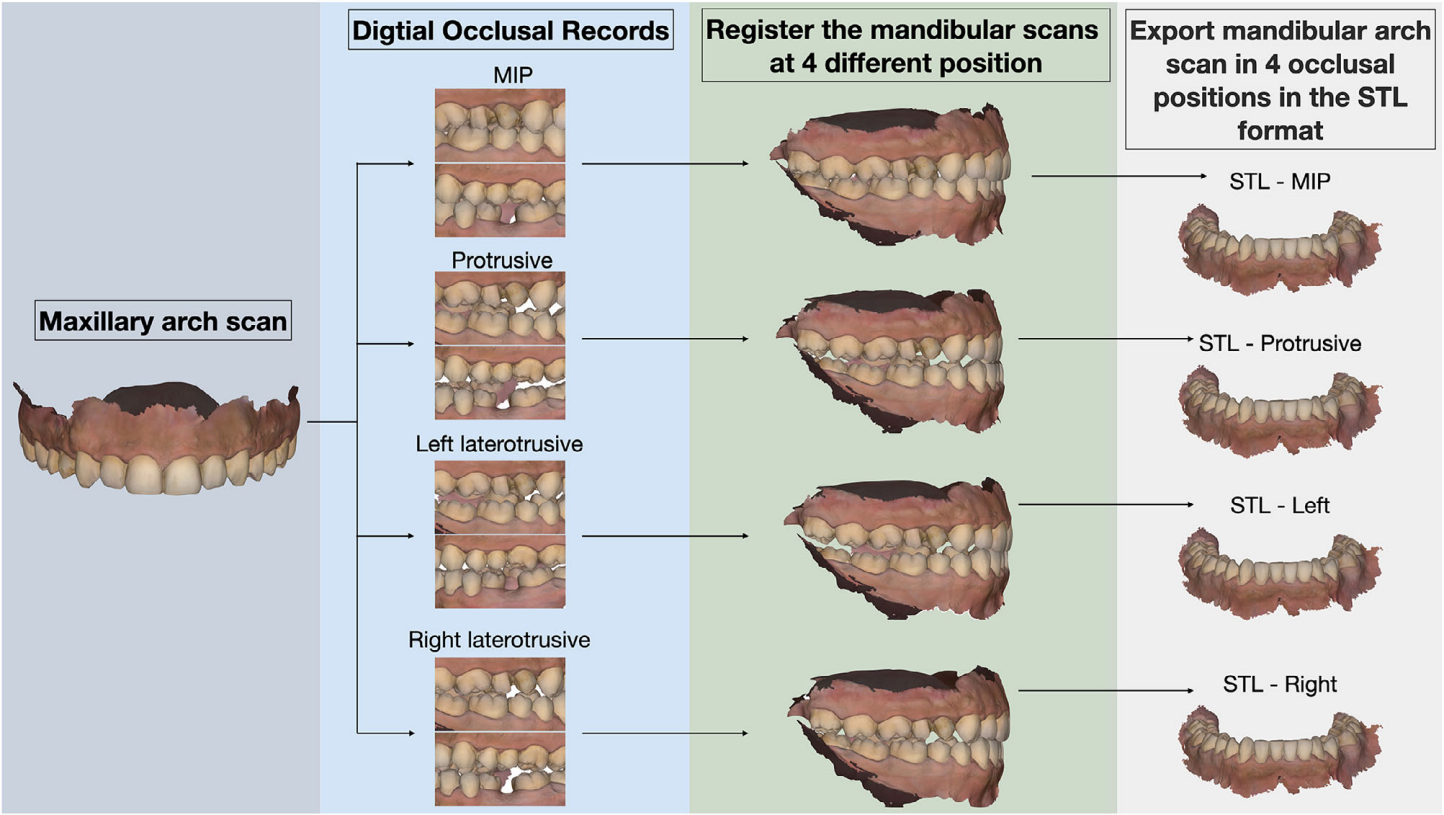
Flow diagram for realigning mandibular arch scans to the proper occlusal position using the maxillary arch scan as the primary registration anchor. Import the digital scans of the maxillary and mandibular arches into CAD software. Register the mandibular arch to the maxillary arch using the maximum intercuspation position (MIP), left laterotrusive, right laterotrusive, and protrusive interocclusal records. Export the mandibular arch scan in STL format in four occlusal positions (MIP, left laterotrusive, right laterotrusive, and protrusive).

**FIGURE 3:**
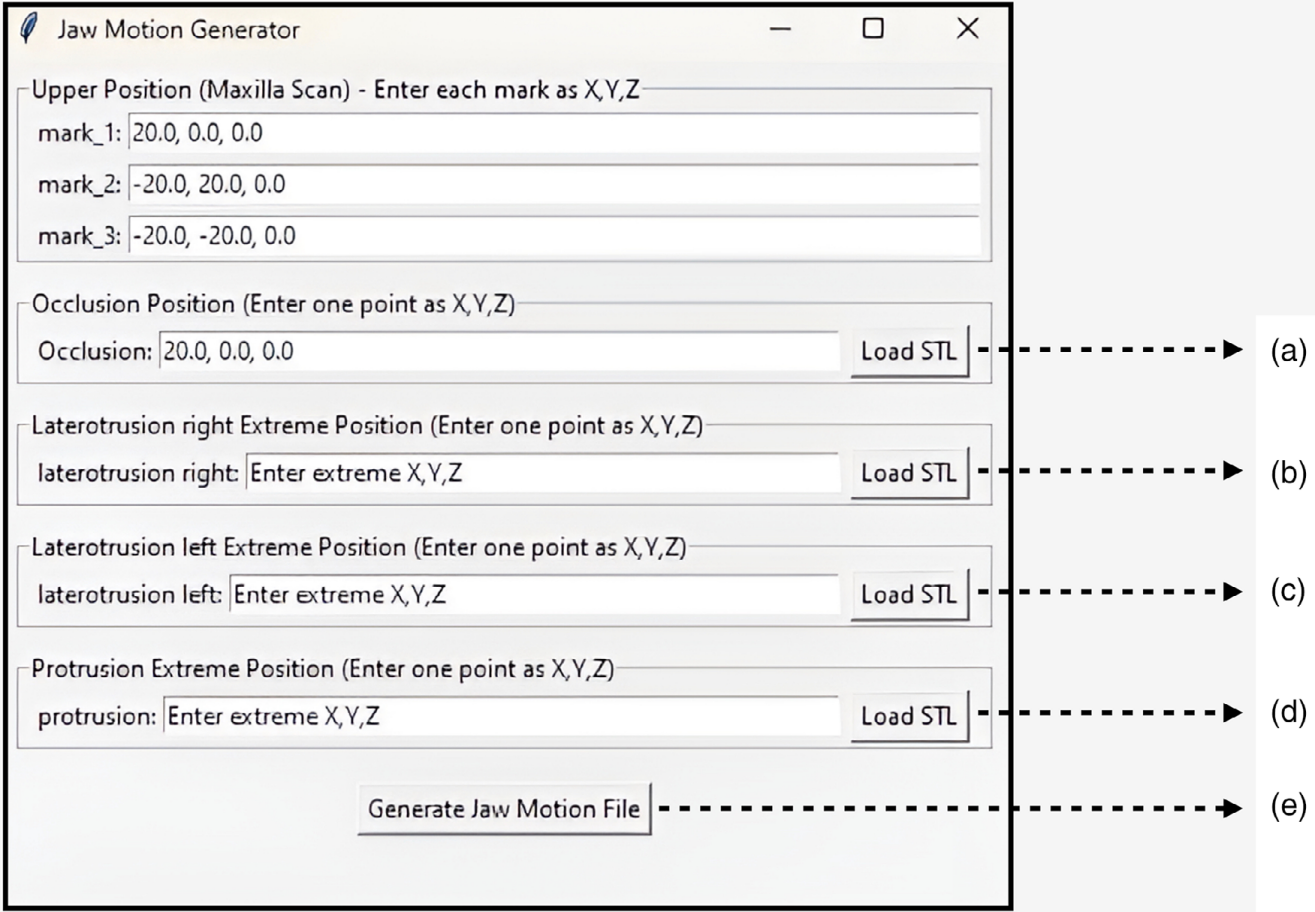
Program interface. Click the “Load STL” icon to load the mandibular arch STL files in different positions: (a) maximum intercuspation position (MIP); (b) right laterotrusive; (c) left laterotrusive; (d) protrusive; (e) after loading the STL files, click the “Generate Jaw Motion File” icon to generate a simulated motion sequence as an Extensible Markup Language (.xml) file. This file is compatible with popular dental CAD software (exocad; exocad GmbH).

**FIGURE 4:**
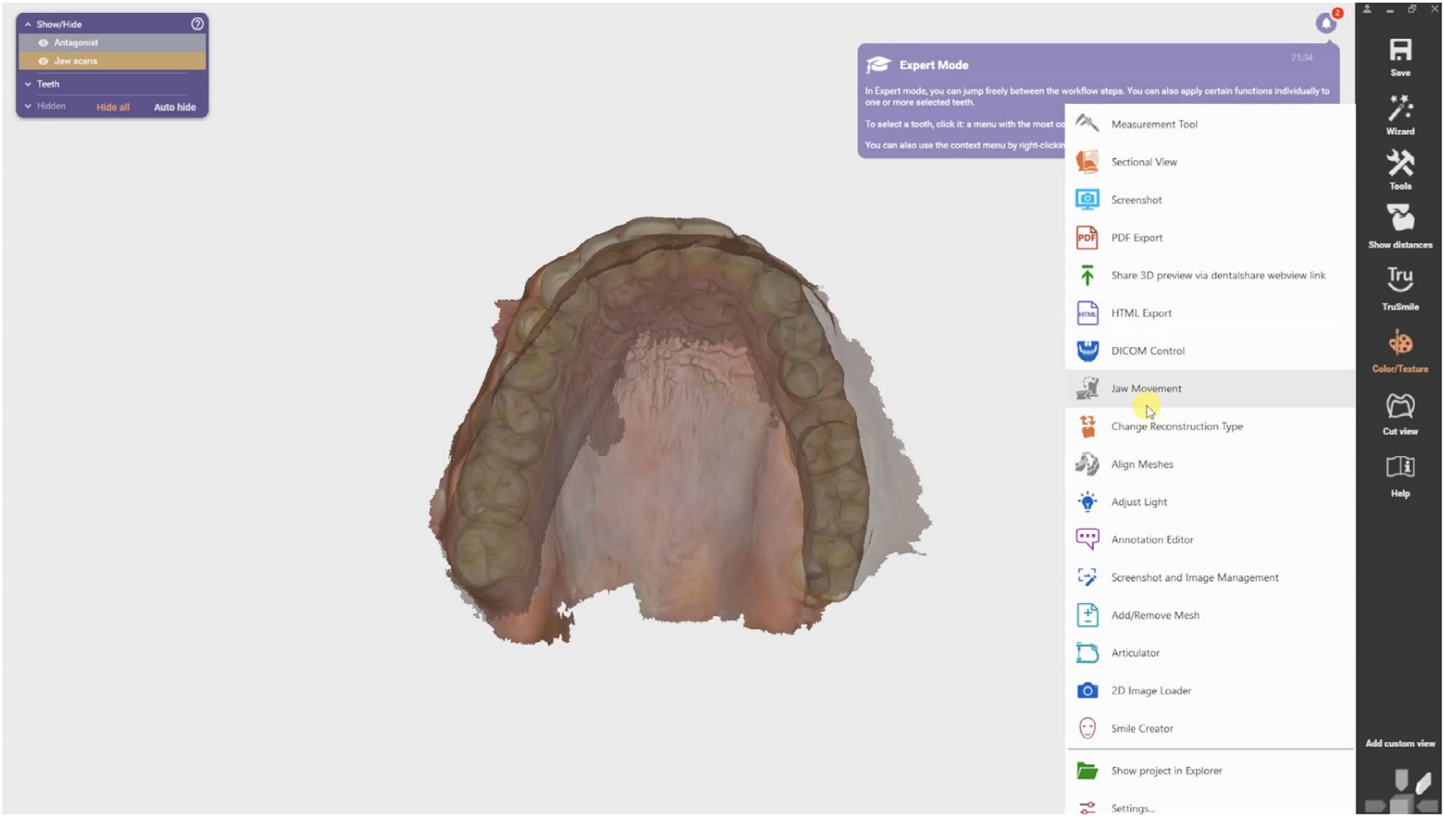
Import the motion file, along with maxillary and mandibular arch scans at the maximum intercuspation position (MIP), using the CAD software.

**FIGURE 5:**
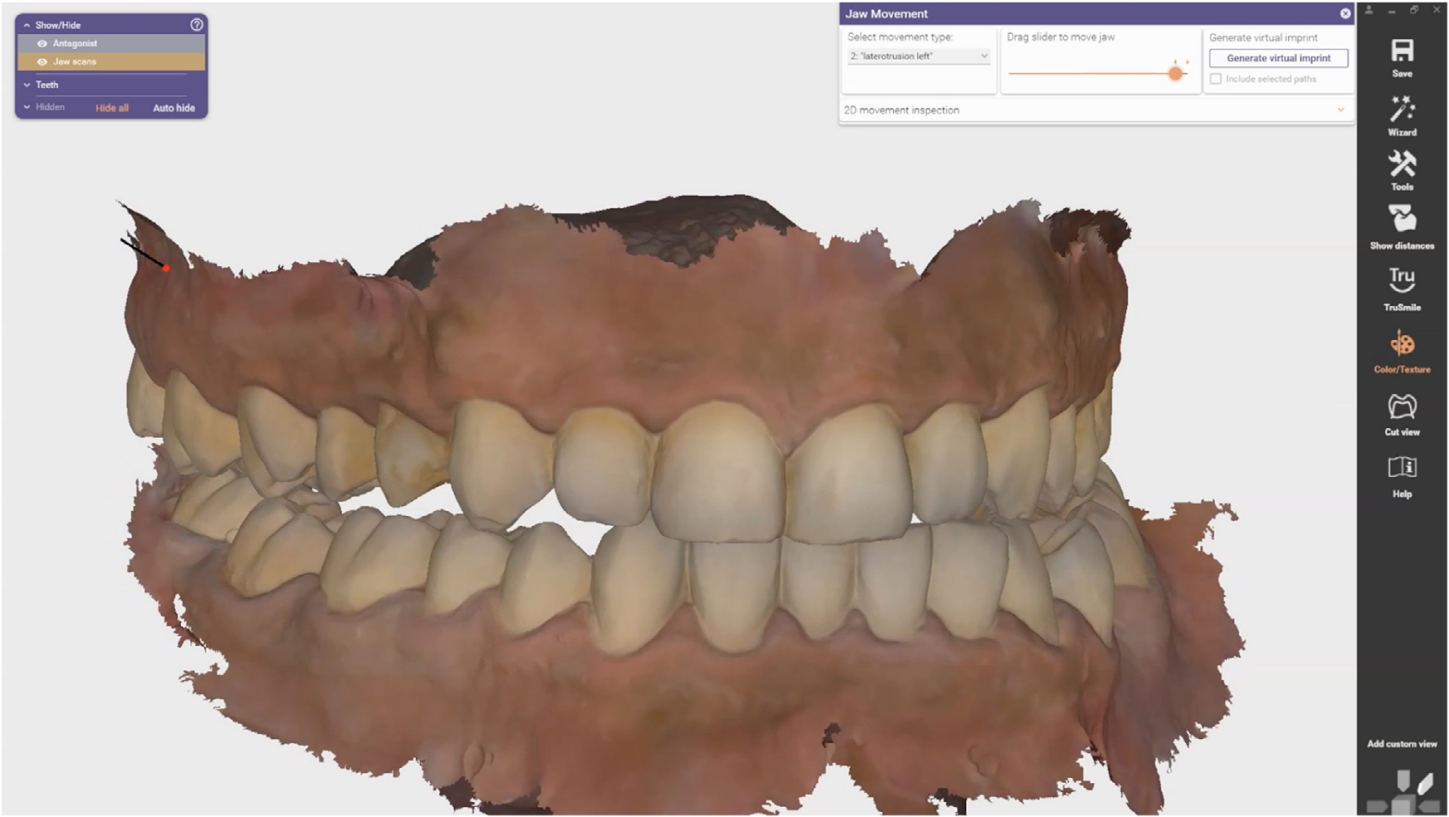
A screen recording of simulated jaw motion is included in Supplementary Material 2. Simulated jaw motion can be visualized in the CAD program and used to design dental prostheses.

1. Capture intraoral scans of the maxillary and mandibular arches using an IOS (TRIOS 4; 3Shape) in accordance with manufacturer protocols. Duplicate the scans in the intraoral scanner's project folder to generate three identical working datasets, including maxillary and mandibular arches.
2. Record a different virtual occlusal relationship in each project copy by capturing interocclusal records in the following positions: maximum intercuspation position (MIP), left laterotrusive position, right laterotrusive position, and full protrusive position (Figure 1).
3. Export the digital scans of the maxillary and mandibular arches in the standard tessellation language (STL) format. Export the interocclusal records (MIP, left laterotrusive, right laterotrusive, and protrusion) in the STL format.
4. Import the digital scans of the maxillary and mandibular arches into CAD software (exocad; exocad GmbH). Register the mandibular arch to the maxillary arch using the MIP interocclusal record. Duplicate the mandibular arch scan three times. Superimpose each duplicate onto the maxillary arch using the left laterotrusive, right laterotrusive, and protrusive interocclusal records. Use the maxillary arch scan as the primary registration anchor and register the mandibular scans in different interocclusal positions relative to the fixed maxillary arch in 3D space (Figure 2). Export the mandibular arch scan in 4 occlusal positions (MIP, left laterotrusive, right laterotrusive, and protrusive positions) in the STL format.
5. Install an open‐source, script‐based programming language program (Python 3.13.2; Python Software Foundation).^16^ Copy, paste, and run the predefined script using the script‐based programming language program (Supplementary Material 1). Follow the program interface to import the mandibular arch scan at 4 occlusal positions (Figure 3a,b,c,d).
6. Allow the software to interpolate dynamic mandibular motion paths between the four recorded static positions using the embedded AI algorithm to generate a continuous motion trajectory that simulates the functional envelope of mandibular movement.
7. Export the simulated motion sequence as an extensible markup language (.xml) file formatted to be compatible with popular dental CAD software (exocad; exocad GmbH) and used in conjunction with the subsequent prosthesis design process (Figure 3e). For specific versions of dental CAD software (exocad; exocad GmbH), change the file extension to  .jawMotion.
8. Import the motion file and maxillary arch scan and mandibular arch scan at MIP position in the CAD software (exocad; exocad GmbH) (Figure 4). Assign the imported motion data to the virtual articulator environment as dynamic occlusion positions. Visualize and verify the motion path during prosthetic design to evaluate functional contacts and excursive guidance (Figure 5 and Supplementary Material 2).

## DISCUSSION

The technique presented introduces a novel scanner‐agnostic approach to simulating patient‐specific dynamic mandibular movement using static occlusal records and a custom AI‐driven script. A key advantage of this method is its accessibility. While some IOS systems may include a proprietary motion capture feature, this is not standard across all devices. This technique is designed to be implemented with any IOS system, democratizing the ability to record patient‐specific motion without requiring a specific, and often costly, hardware ecosystem. Unlike commercial jaw tracking systems that rely on expensive equipment and real‐time motion capture hardware, this technique leverages an intraoral scanner and static records of mandibular positions at key functional extremes, namely MIP, protrusive, and right and left laterotrusive.

A central feature of this workflow is its openness and flexibility. This openness extends to this technique's most significant clinical and technical distinction: data interoperability. The dynamic motion data captured by closed commercial systems is typically intended for use strictly within their proprietary software. This closed‐architecture approach prevents the functional data from being utilized in third‐party CAD software, severely limiting the clinician's or technician's choice of design and manufacturing tools. In contrast, this technique is designed to generate data in an open, interoperable format (.xml), granting users the freedom to integrate patient‐specific dynamics into any compatible downstream software platform. The predefined script (Supplementary Material 1) can be developed using open‐source platforms that support Python programming. For instance, Jupyter Notebook (Project Jupyter) offers a web‐based interactive computing environment for creating and sharing documents that contain live code, equations, visualizations, and narrative text.^17^ Additionally, Google Colab (Google) is a hosted Jupyter service that requires no local setup and offers free, quota‐limited compute, widely used in machine learning, data science, and education.^18^

In Step 4, the mandibular arch scans had to be re‐registered in the CAD software using the maxillary arch scan as the primary registration anchor. This was necessary because the intraoral scanner initially registered the maxillary arch to the mandibular arch (using the mandibular arch as the primary anchor) at different interocclusal positions. This misalignment would cause the generated motion to appear as movement of the maxillary arch instead of the mandibular one. Therefore, re‐registration of the mandibular arch scans in the CAD software was required. Due to the limitations of the script‐based programming language, which only processes STL files, only the re‐registered mandibular arches were exported as STL files to generate the motion file. Each mandibular position was registered against the same maxillary reference, ensuring all mandibular datasets were spatially normalized.

Once aligned, a predefined script (Supplementary Material 1) employed an AI‐based interpolation algorithm to estimate the intermediate positions of the mandible across the excursive envelope of motion. The interpolation process utilized a quantified point cloud system, where specific landmarks or mesh vertices on the mandibular STL models were tracked throughout the motion sequence. The algorithm analyzed the spatial transformations between corresponding points at different occlusal positions and calculated transitional vectors. These vectors were then used to generate a series of interpolated positions between maximum MIP and each excursive endpoint, resulting in smooth, time‐sequenced mandibular trajectories. Typically, the interpolation generates a user‐defined number of transitional frames, referred to as quantified points, between each static record. For example, in this report, a 100‐point interpolation between MIP and protrusion produced 100 intermediate STL positions representing progressive mandibular advancement. The final output is a continuous mandibular motion path, exported as an Extensible Markup Language (.xml) file. This format is compatible with a major dental CAD platform that supports virtual articulators or jaw motion modules (Figures 4 and 5).

**FIGURE 6:**
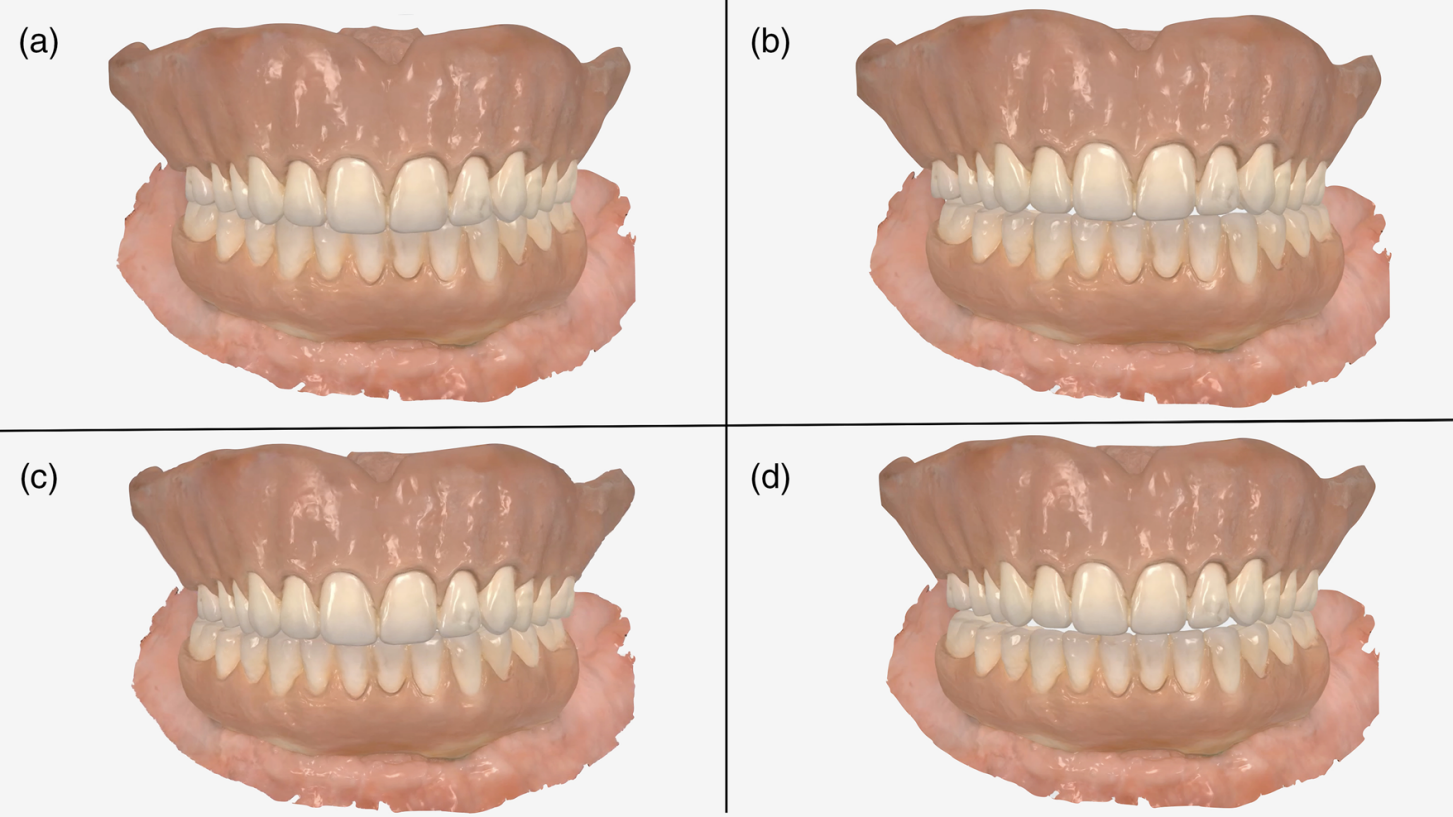
Intraoral scans of the maxillary interim complete denture and mandibular implant‐supported interim prosthesis were duplicated three times and registered at different interocclusal positions: (a) centric occlusion; (b) left excursion; (c) right excursion; (d) protrusion.

**FIGURE 7:**
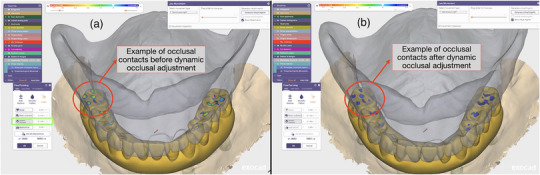
Example of dynamic occlusal adjustment in the design software (exocad; exocad GmbH): (a) occlusal contacts before applying the dynamic occlusal adjustment function; (b) after dynamic adjustment, occlusal contacts demonstrate improved contact distribution based on the specified dynamic occlusal clearance, as indicated in the green highlight box (dynamic occlusion set at 0.1 mm for demonstration).

While the primary example in this report features a dentate patient, the method's application extends to complex rehabilitations. Its core strength lies in its ability to simulate motion at a predetermined occlusal vertical dimension (OVD). For instance, in cases of edentulism or severe wear requiring an altered OVD, the clinician first establishes and verifies the new OVD using conventional methods, such as occlusal rims, gothic arch tracing, custom jigs, trial prostheses, or interim prostheses. With the OVD physically maintained by these devices, the digital scanning procedure is then performed to capture the centric occlusion (CO) and border positions (protrusive, right laterotrusive, and left laterotrusive positions). The workflow was applied to an edentulous patient restored with a maxillary complete denture and a mandibular implant‐supported interim prosthesis to demonstrate this principle. A patient‐specific dynamic simulation can be generated by capturing the static border positions and the CO position (which replaces MIP in such cases) at the planned vertical dimension (Figure 6 and Supplementary Material 3). After generating the jaw motion file, it can be imported into the design software (exocad; exocad GmbH) to verify dynamic occlusal contacts on the digital wax‐up. Clinicians and technicians can assess dynamic occlusion in real time before finalizing prosthetic design and manufacturing. This provides an efficient method to minimize chairside occlusal adjustments and optimize functional harmony. A video demonstration of this application is provided (Supplementary Material 4), together with a representative figure showing the automatic dynamic occlusal adjustment function in the digital design software by using the jaw motion file (Figure 7). In this example, the workflow was applied to the design of a mandibular definitive implant‐supported prosthesis. Regardless of the type of prosthesis, digital records can be obtained by following the procedure described in this technique. A jaw motion file can then be generated and incorporated into the digital design process, as long as a digital wax‐up is completed, to allow dynamic design adjustment.

While the technique provides functional insight, it has some limitations. The AI‐based interpolation generates a simplified trajectory between static points and, as such, does not capture the complex, nonlinear nature of true mandibular movement. It currently cannot account for the specific curvature of the condylar pathway, rotational asymmetries, or neuromuscular dynamics, which may be critical in patients with temporomandibular disorders. Furthermore, the quality of the simulation is inherently dependent on the precision of the static occlusal records, as any inaccuracies in capturing the excursive endpoints will be propagated throughout the interpolated path. From a clinical standpoint, the technique requires capturing extra interocclusal positions, which adds to the overall chair time. The predefined script provided in this report (Supplementary Material 1) is currently compatible only with a single CAD software (exocad; exocad GmbH), and rigorous validation studies comparing this AI simulation to physical jaw tracking systems are essential to quantify its accuracy and establish its clinical reliability.

## SUMMARY

A novel digital technique was developed to simulate dynamic mandibular movement using static virtual occlusal records captured in MIP, protrusive, and lateral excursive positions. The method employs a custom‐developed, Python‐based script to interpolate functional motion paths, which are then exported as a CAD‐compatible  .xml jaw motion file. This technique enables the integration of simulated dynamic occlusion into CAD software without requiring specialized jaw‐tracking hardware. The scanner‐agnostic workflow provides an accessible alternative for clinicians and technicians involved in designing prostheses where excursive dynamics are critical to treatment success.

## Supporting information




**Supplementary Material 1**. A predefined script file used for programming in the script‐based programming language program (Python 3.13.2; Python Software Foundation).

**Supplementary Material 2**. Screen recording of jaw motion simulation.

**Supplementary Material 3**. Screen recording of jaw motion simulation for clinical example #2, maxillary interim complete denture and mandibular implant‐supported interim prosthesis.

**Supplementary Material 4**. Screen recording demonstrating the application of dynamic occlusal adjustment during the digital design process (exocad; exocad GmbH). The video illustrates how the jaw motion file can be used to evaluate and refine occlusal contacts in real time on a digital wax‐up before finalizing prosthetic design and manufacturing.
